# Relationship between the Expression of Matrix Metalloproteinase and Clinicopathologic Features in Oral Squamous Cell Carcinoma

**Published:** 2015-05

**Authors:** Amir Hossein Jafarian, Leila Vazife Mostaan, Nema Mohammadian Roshan, Kamran Khazaeni, Shafagh Parsazad, Hamed Gilan

**Affiliations:** 1 *Cancer Molecular Research Center, Ghaem Hospital, Faculty of Medicine, Mashhad University of Medical Sciences, Mashhad, Iran.*; 2 *Solid Tumour Treatment Research Center, Omid Hospital, Faculty of Medicine, Mashhad University of Medical Sciences, Mashhad, Iran.*; 3 *Departmentof Anatomicoclinical Pathology, Ghaem Hospital, Faculty of Medicine, Mashhad University of Medical Sciences Mashhad, Iran.*

**Keywords:** Lymphatic metastasis, Matrix metalloproteinase, Oral squamous cell carcinoma

## Abstract

**Introduction:**

Squamous cell carcinoma of the oral cavity is one of the most important and common types of head and neck malignancy, with an estimated rate of 4% among all human malignancies. The aim of this study was to determine the association between expression of matrix metalloproteinase 2 and 9 and the clinicopathological features of oral squamous cell carcinoma (OSCC).

**Materials and Methods:**

One hundred existing samples of formalin-fixed paraffin embedded specimens of OSCC were evaluated by immunohistochemistry staining for matrix metalloproteinase 2 and 9 antibodies. Samples were divided into four groups: negative, <10%, 10–50%, and >50%. Patient records were assessed for demographic characteristics such as age and gender, smoking and family history of OSCC as well as tumor features including location, differentiation, stage and lymph node involvement.

**Results:**

In this study, 58 patients (58%) were male and 42 (42%) female. The mean age of patients was 60.38±14.07 years. The average number of lymph nodes involved was 8.9±3.8. Tumoral grade, tumoral stage, lymphatic metastasis and history of smoking were significantly related to MMP2 and MMP9 expression.

**Conclusion:**

Our study demonstrated that MMP2 and MMP9 expression are important in the development of OSCC.

## Introduction

Squamous cell carcinoma (SCC) accounts for more than 90% of all head and neck malignancies. In the United States, head and neck squamous cell carcinomas (HNSCC) comprise about 4% of all cancers (1). The 5-year survival rate for SCC is estimated at approximately 60% (1). Local invasion of tumoral cells and regional lymph node involvement leads to a poor prognosis in HNSCC. Magnitude of conflicts and HNSCC stage are determining factors in choosing therapeutic options (2). 

Matrix metalloproteinases (MMPs ) are an important category of endopeptidases and are vital for tissue remodeling, extracellular matrix (ECM) degradation and cellular migration (3). In a normal physiological situation, MMPs are not over-expressed. MMP expression is controlled by hormones, cytokines and growth factors. Some physiologic processes in which MMPs play a crucial role are angiogenesis, apoptosis, bone remodeling, embryonic development, endometrial cycling, immune response, and nerve growth, for example (4).

Metalloproteinase inhibitors (MMPIs) and tissue inhibitors of matrix metalloproteinase (TIMPs) down-regulate MMP over-expression (4,5). MMPs were first noted as tumor progression-accelerating enzymes (6). MMPs are involved in pathologic processes such as multiple sclerosis, skin ulceration, nephritis, vascular disease, and metastasis, among others (3). MMPs are classified in eight structural classes, three of which are membranous (7). It is estimated that MMPs are responsible for almost all human cancers, and their expression correlates with poor outcomes (8). Some MMPs such as MMP7 are synthesized by tumoral cells while others such as MMP2 and both endothelial and inflammatory cells express MMP9 (9).

MMPs are essential in cancer development and influence cell migration, cell growth, colony formation, angiogenesis, and local invasion (10). Recent studies and clinical trials have focused on producing synthetic MMP inhibitors. Cyclophosphinamide, cyclophosphonamide-based hydroxamic acids, biphenylsulfonamide carboxylic acids, caffeoyl pyrrolidine derivatives, sulfonamide hydroxamates and nonhydroxamates are some examples of MMP inhibitors (6). Regional lymph node involvement and metastases are factors associated with poor prognosis. 

The aim of this study was to determine the association between expression of MMP and clinicopathologic features in OSCC.

## Materials and Methods

This was a cross- sectional study. After University Ethics Committee approval, 100 patients with OSCC from two hospitals (Ghaem, Emam Reza) in Mashad between the years 2004–2008 were recruited and followed for a minimum of 5 years. Patient records were assessed for demographic characteristics such as age and gender, smoking and family history of OSCC as well as tumor features including location, differentiation, stage, and lymph node involvement. 

**Fig1:**
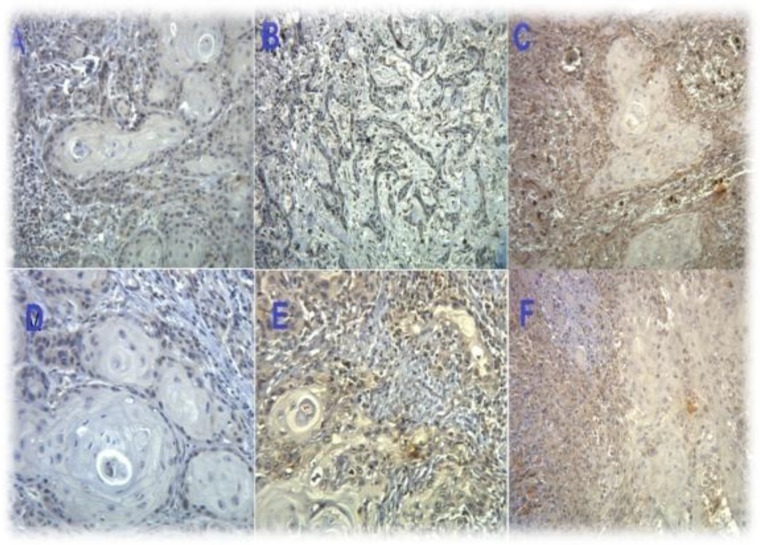
Representative immunohistological manifestations of mild (A), moderate (B), and severe (C) MMP9 expression and mild (D), moderate (E), and severe (F) MMP2 expression in oral squamous cell carcinoma. MMPs were particularly expressed on the tumoral cell membrane surface and cytoplasm

Tissue samples had been provided by surgery or biopsy before adjuvant treatment and stored in pathology departments. Formalin-fixed OSCC specimens which were embedded in paraffin during the pretreatment phase were sectioned at a thickness of 5 µm. For the deparaffinization phase, samples were placed for approximately 10 minutes in a methanol plate which contained hydrogen peroxide. To block non-specific antigens, albumin and rabbit polyclonal antibodies against MMP2 and MMP9 (Dako Laboratory Inc, Denmark) were used and this mixture was incubated at 4°C for 15 hours. Slides were then washed with buffered phosphate solution. Immunohistochemical assessment was performed by avidin- biotin complex and cytoplasmic staining percentage was counted in tumor and non-tumor cells. The bone marrow sample was used as a positive control. All samples were evaluated by two pathologists who were blinded with respect to clinical manifestation, OSCC stage and patient outcome. Based on the degree of immunohistochemical staining, cells were divided into four groups: negative, <10%, 10–50%, and >50% (Fig.1). A digital image was captured to resolve disagreements and to make a final decision. All samples of normal tissues showing dysplasia had positive results. The sample size in this study was calculated using available hospital pathologic records, and there was no practical bias in the study.

Data were analyzed using SPSS software. The chi–square was used for data analysis. The significance level was considered less than 0.05. 

## Results

In total, 58 patients (58%) were male and 42 (42%) were female. Patients ranged from 20 to 84 years of age, with a mean age of 60.5±14.07 years (Table.1). A history of smoking and tobacco use was noted in 39 and four cases, respectively. A family history of OSCC was found in five patients. Surgical removal of a tumor with or without adjuvant chemotherapy or radiotherapy was performed in 76 and 22 cases, respectively. The average number of lymph nodes involved was 8.9±3.8. Seventy-six samples were located in the tongue, 10 in the buccal mucousa,10 in the mandibule, two in the mouth, and three in the lip. Sixty-seven samples were well differentiated, 31 were moderately differentiated, and two were poorly differentiated. Relapse occurred in 41 patients. 

**Table 1:** Patient demographic characteristics

		**No/ %**
Gender	Male	58
	Female	42
Age	<60	42
	≤60	58
Differentiation	Well	67
	Moderate	31
	Poor	2
Smoking	-	61
	+	39
Family history of SCC	-	95
	+	5
Stage	1	35
	2	39
	3	14
	4	12
Lymph node involvement	-	67
	+	33
	-	59
Relapse	Local	36
	Regional	5

There was no relationship between gender or age with respect to MMP expression. Patients with a positive history of smoking showed greater MMP2 and MMP9 expression (P=0.004, P=0.027, respectively). MMP2 and MMP9 expression was statistically associated with a number of tumor features such as location (P=0.002), tumoral differentiation, tumoral stage and tumoral relapse. Over-expression of MMP2 was related to lymph node involvement (P=0.021), but MMP9 expression was not higher in patients with lymph node involvement. Expression of MMPs was higher in patients who had a mandible tumor (Table.2).

**Table 2:** Association between MMP2 and MMP9 expressions and clinicopathologic features

		**MMP2 (N/%)**					**MMP9 (N/%)**				**P value**
		0	1	2	3		0	1	2	3	
Gender	Male	24(58.5)	9(60)	16(57.1)	9(56.3)	0.906	27(55.1)	16(64)	10(58.8)	5(55.6)	0.904
	Female	17(41.5)	6(40)	9(41.9)	4(43.8)		22(44.9)	9(46)	7(41.2)	4(44.4)	
Age	60>	19(46.3)	6(40)	13(46.4)	4(25)	0.482	22(44.9)	10(40)	5(22.4)	5(55.6)	0.574
	60<	22(53.7)	9(60)	15(53.6)	12(75)		27(55.1)	15(60)	12(70.6)	4(44.4)	
Tumor location	Tongue	37(60)	12(16)	15(16)	11(8)	0.002	45(60)	12(16)	12(16)	6(8)	0.001
	Buccal	1(10)	1(10)	5(50)	3(30)		1(10)	6(60)	1(10)	2(20)	
	Mandible	.	.	7(77.8)	2(22.2)		.	7(77.8)	1(11.1)	1(11.1)	
	Mouth	2(100)	.	.	.		2(100)	.	.	.	
	Lip	1(100)	2	.	.		1(33.3)	.	2(66.6)	.	
	Buccal and Mandible	.	.	1(100)	.		.	.	1(100)	.	
Smoking	No	26(42.6)	11(18)	10(16.4)	14(23)	0.004	3(54.1)	13(21.3)	9(14.8)	6(9.8)	0.027
	Yes	15(38.5)	4(10.3)	18(46.2)	2(5.1)		16(41.0)	12(30.8)	8(20.5)	3(7.7)	
OSCC history	No	41(44.1)	13(14.0)	26(28.9)	13(14.0)	0.023	8(8.3)	13(14.1)	1(25)	47(50.5)	0.005
	Yes	0	0	2(40)	3(60)		1(20)	4(80)	25(26.9)	.	
Differentiation	well	30(44.8)	13(19.4)	20(29.8)	4(6)	0.001	38(56.7)	19(28.4)	7(10.4)	3(4.5)	0.004
	Moderate	11(35.5)	2(6.5)	8(25.8)	10(32.3)		11(35.5)	6(19.4)	10(32.3)	4(12.9)	
	Poor	.	0	.	2(100)		.	.	.	2(100)	
Stage of tumor	Stage 1	20(57.1)	9(25.3)	5(14.3)	1(2.9)	0.001	26(74.3)	4(11.4)	4(11.4)	1(2.9)	0.001
	Stage 2	17(43.6)	5(12.8)	15(38.5)	2(5.1)		17(43.6)	17(43.6)	3(7.7)	2(5.1)	
	Stage 3	4(28.6)	1(7.1)	6(42.9)	3(21.4)		6(42.9)	4(28.6)	3(21.4)	2(7.1)	
	Stage 4	.	0	2(16.7)	10(83.3)		.	.	7(58.3)	5(41.7)	
Lymph node involvement	-	24(35.8)	15(22.4)	19(28.4)	9 (13.4)	2(0.08)	31(46.3)	21(31.3)	10(14.9)	5(7.5)	1(0.20)
	+	17(51.5)	.	9(27.3)	7(21.2)		18(54.5)	4(12.1)	7(21.2)	4(12.1)	

## Discussion

The expression of MMP in non-tumor tissues is low. In tumor tissue, the over-expression of MMP is due to growth factors and cytokines (11,12).

The de Vicente study showed the association between MMP2- and MMP9-expression and lymph node involvement (11). Our results also confirmed that over-expression of MMP2 was related to lymph node involvement (P=0.021), but that MMP9 expression was not higher in patients with lymph node involvement. This finding corroborates the role of MMP2 in tumor invasion and metastasis. In vitro and in* vivo* research has shown that MMP2 over-expression is necessary for tumor invasion and that MMP2 and MMP9 promote tumoral cell colony formation (12,13). 

Results of the Mashhadi study were consistent with our findings with respect to the association between MMP expression and histopathological grade in OSCC patients (14). In some studies, correlation between alcohol consumption (not common in our country) and over-expression of MMP has been proven (15). Past research has also shown that smokeless tobacco is a risk factor for OSCC, and in our study all patients who had used smokeless tobacco had over-expression of MMP2 and MMP9. 

Our finding confirmed that over-expression of MMPs had not correlated with gender or age, and could therefore be a reliable index in all patients. On the other hand, MMP2 and MMP9 expression were higher in patients with a positive family history of SCC (P<0.05) and all of the individuals with a SCC-positive family history had negative MMP2 and MMP9. This suggests a role for MMP2 and MMP9 as a screening test in patients who are at high risk of OSCC. 

Expression of MMPs was higher in patients who had a mandible tumor. Poor tumoral differentiation was accompanied with higher MMP2 and MMP9 expression. Patients with stage IV OSCC had over-expression of MMPs. Moreover, death tended to occur in patients with over-expression of MMP2 and MMP9. 

Our study shows that there was a significant association between MMP2 over-expression and local or regional relapse of OSCC. This relationship could be explained by the angiogenesis-regulating effect of MMP2 and its role in endothelial cell migration. The Fang study confirmed that MMP2 down-regulation reduces tumoral angiogenesis (16). 

## Conclusion

Our study demonstrated that MMP2 and MMP9 expression are important in the development of OSCC patients. 
